# Quantification of guanidine in environmental samples using benzoin derivatization and LC-MS analysis

**DOI:** 10.1016/j.mex.2024.102972

**Published:** 2024-09-24

**Authors:** Richard Gruseck, Marton Palatinszky, Michael Wagner, Thilo Hofmann, Michael Zumstein

**Affiliations:** a Centre for Microbiology and Environmental Systems Science, Department of Environmental Geosciences, University of Vienna, Josef-Holaubeck-Platz 2, 1090 Vienna, Austria; b Doctoral School in Microbiology and Environmental Science, University of Vienna, Djerassiplatz 1, 1030 Vienna, Austria; c Centre for Microbiology and Environmental Systems Science, Division of Microbial Ecology, University of Vienna, Djerassiplatz 1, 1030 Vienna, Austria; d Center for Microbial Communities, Department of Chemistry and Bioscience, Aalborg Univiersity, Fredrik Bajers Vej 7H, 9220 Aalborg, Denmark

**Keywords:** Guanidino compounds, Environmental and biological matrices, Urine, Liquid chromatography coupled to mass spectrometry, Quantification of pre-column derivatized guanidine with LC-MS/MS

## Abstract

The recent discovery of guanidine-dependent riboswitches in many microbes raised interest in the biological function and metabolism of this nitrogen-rich compound. However, very little is known about the concentrations of guanidine in the environment. Several methods have been published for quantifying guanidine and guanidino compounds in human urine and blood, often relying on derivatization followed by fluorescence detection. We adapted this analytical approach using benzoin as the derivatization agent to sensitively and selectively quantify guanidine in environmental samples, thereby facilitating future research on the biological and environmental roles of guanidine. This adapted method was applied to human urine, raw wastewater, and biological growth media as relevant matrices. Our liquid chromatography-tandem mass spectrometry analyses of the derivatized solutions identified a different major derivatization product than previously reported. This product was consistently observed across various substrates (guanidine, methylguanidine, and arginine) and derivatization agents (benzoin and anisoin). We observed a constant background signal, restricting our analyses to a lower limit of quantification of 50 nM. Despite this limitation, our method allowed for the quantification of guanidine concentrations significantly lower than those reported in previous derivatization-based studies.

• Selective and sensitive detection of guanidine by LC-MS.
• Method development and validation for robust detection of guanidine in environmental samples.
• Reduction of sample preparation steps and reduced usage of toxic chemicals compared to previous methods.

## Graphical abstract

**Figure:**
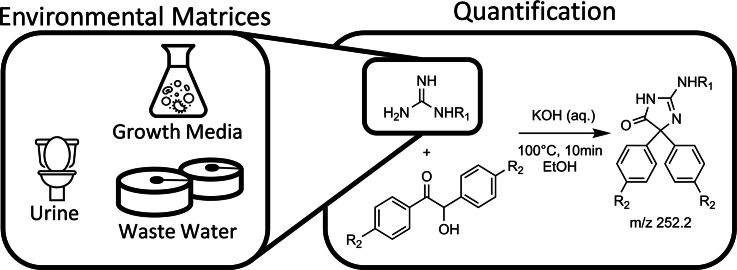

Specifications table

**Table:** 

Subject area:	Environmental Science
More specific subject area:	Environmental Analytical Chemistry
Name of your method:	Quantification of pre-column derivatized guanidine with LC-MS/MS
Name and reference of original method:	Kai, M.; Miura, T.; Kohashi, K.; Ohkura, Y. New Method for the Fluorimetric Determination of Guanidino Compounds with Benzoin. *Chemical & Pharmaceutical Bulletin***1981**, *29* (4), 1115–1120.
Resource availability:	The resources necessary to reproduce our method are provided in this article.

## Background

Guanidine has recently received increased attention from the biological and environmental research communities [1,2]. For example, the discovery of guanidine-selective bacterial riboswitches (i.e., RNA that act as ligands to metabolites and regulate gene expression) in many microbes suggested an important role of guanidine in bacterial metabolism [3,4]. More recently, it was shown that guanidine can enable growth of complete ammonia oxidizers without the need for other sources of energy, nitrogen, or reductants [5]. Based on these studies, we foresee an increase in research into the biological function and metabolism of guanidine, as well as in its abundance in various environmental systems. To facilitate such investigations, we developed a method based on derivatization and subsequent analysis by liquid chromatography coupled to mass spectrometry (LC-MS). Crucially, this method is applicable over a wide dynamic concentration range and across different environmental and biological matrices. Previously, guanidine quantification has been primarily limited to human urine using derivatization with benzoin and fluorescence detection [1]. Our objective was to adapt these methods to a sensitive triple-quadrupole mass spectrometry-based approach. This article supplements the related article by presenting method validation data including sample stability data, calibration curves in different matrices, and discussing limitations of measuring guanidine at trace levels. Additionally, we demonstrate the versatility of our detection method for different derivatization agents and other guanidino compounds [5].

## Method details

### Chemicals and materials

Guanidine hydrochloride (≥ 99 %, article number: G3272), methylguanidine hydrochloride (98 %, 222,402), benzoin (≥ 99 %, 8.01776), l-arginine (> 99.5 %, 11,009), potassium hydroxide (≥ 85 %, 1.05033), ethanol (≥ 99.8 %, 02,851), formic acid (≥ 98 %, 5.43804), β-mercaptoethanol (≥ 99 %, 8.05740) and sodium sulfite (≥ 98 %, 239,321) were purchased from Sigma-Aldrich. Hydrogen chloride solution (32 %, 20,254.321) and acetonitrile (≥ 99.9 %, 20,060.320) were purchased from VWR. Anisoin (97 %, B21559.14) was purchased from Thermo Fisher Scientific. 2-Methoxyethanol (> 99.5 %, 149,360,010) was purchased from Across Organics. Purified water was obtained from a water purification system (0.071 µS/cm, Elga Veolia, PURELAB Chorus). Protein LoBind Tubes (0.5 mL: 0,030,108,434) and Safe-Lock Tubes (0.5mL: 0,030,121,023; 1.5 mL: 0,030,120,086) were purchased from Eppendorf. Glass HPLC vials (amber, 1.5 mL, BA10214) and caps (Silicone/PTFE, BA10074) were purchased from Bruckner Analysentechnik. Plastic HPLC vials (QuanRecovery MaxPeak Polypropylene 300 µL, 186,009,186) and caps (pre-slit Silicone/PTFE, 186,005,827) were purchased from Waters.

### Derivatization protocol

If not otherwise specified, guanidine was derivatized according to the following protocol: 150 µL of an aqueous sample was cooled to 0 °C in a 0.5 mL Protein LoBind tube and spiked with 75 µL of a benzoin solution in ethanol (4 mM), 75 µL of water, and 150 µL of an aqueous potassium hydroxide solution (1.6 M). The solution was mixed by manually inverting the tube three times. For the derivatization, the tubes were added to a stirred boiling water bath (a maximum of 32 tubes in parallel). After 10 min, the tubes were cooled in an ice bath for 2 min. Subsequently, 25 µL of an aqueous hydrogen chloride solution (4.8 M) was added. The resulting solution was mixed and transferred to a 1.5 mL Safe-Lock tube and centrifuged at 10′000 g for 2 min. The supernatant was transferred to a glass HPLC vial and stored at −20 °C until quantification by HPLC-MS/MS. Supernatants of samples with a guanidine concentration greater than 5 µM were subsequently diluted with water to reach a maximal guanidine concentration of 5 µM.

### HPLC-MS analyses

Derivatization solutions were analyzed with high-performance liquid chromatography (HPLC, Agilent 1290 Infinity II) coupled to triple quadrupole mass spectrometer (MS/MS, Agilent 6470). We used an InfinityLab Poroshell 120 Bonus-RP (Agilent, 2.7 µM, 2.1 × 150 mm) column for separation, an injection volume of 2 µL, a flow rate of 0.4 mL/min, a column compartment temperature of 40 °C, and the following eluents: (A) Purified water + 0.1 % (v/v) formic acid, (B) acetonitrile + 0.1 % (v/v) formic acid. The eluent gradient was as follows: 0–1.5 min: 5 % B, 1.5–4 min: 5–61 % B, 4–4.5 min: 61–95 % B, 4.5–7 min: 95% B, 7–8 min: 95–5 % B, 8–10 min: 5 % B. The MS source parameters were set as follows: positive mode electrospray ionization, gas temperature: 250 °C, gas flow: 10 L/min, nebulizer: 45 psi, sheath gas temperature: 280 °C, sheath gas flow: 11 L/min, capillary voltage: 3.5 kV, nozzle voltage: 0.5 kV. The LC flow was diverted to waste between 0–3 min and 5–10 min to reduce deposits in the ESI source. At a retention time of 3.75 min, the following product ions of the parent ion at 252.2 *m/z* were monitored: 182.1 *m/z* (quantifier) and 104.1 *m/z* (qualifier) with a ratio of 1:0.68.

For product identification, we used an UHPLC (Thermo Scientific Dionex Ultimate 3000) coupled to a Thermo Scientific Q Exactive mass spectrometer - with the same chromatography method as described above. The MS parameters were set as follows: MS full-scan: range: 100–1500 *m/z*, resolution: 140 000, AGC target: 10^6^, Maximum IT: 100 ms, positive electrospray ionization (tune data: capillary temperature: 275 °C, sheath gas: 15, aux gas: 10, sweep gas: 1, S-lens RF: 50.0), MS/MS acquisitions: Top5, resolution: 17 500, AGC target: 10^5^, Maximum IT: 50 ms, isolation window: 1.0 *m/z*, NCE (stepped): 10, 20, 30, dynamic exclusion time: 2.0 s.

## Method validation

### Characterization of derivatization products

**Fig. 1:**
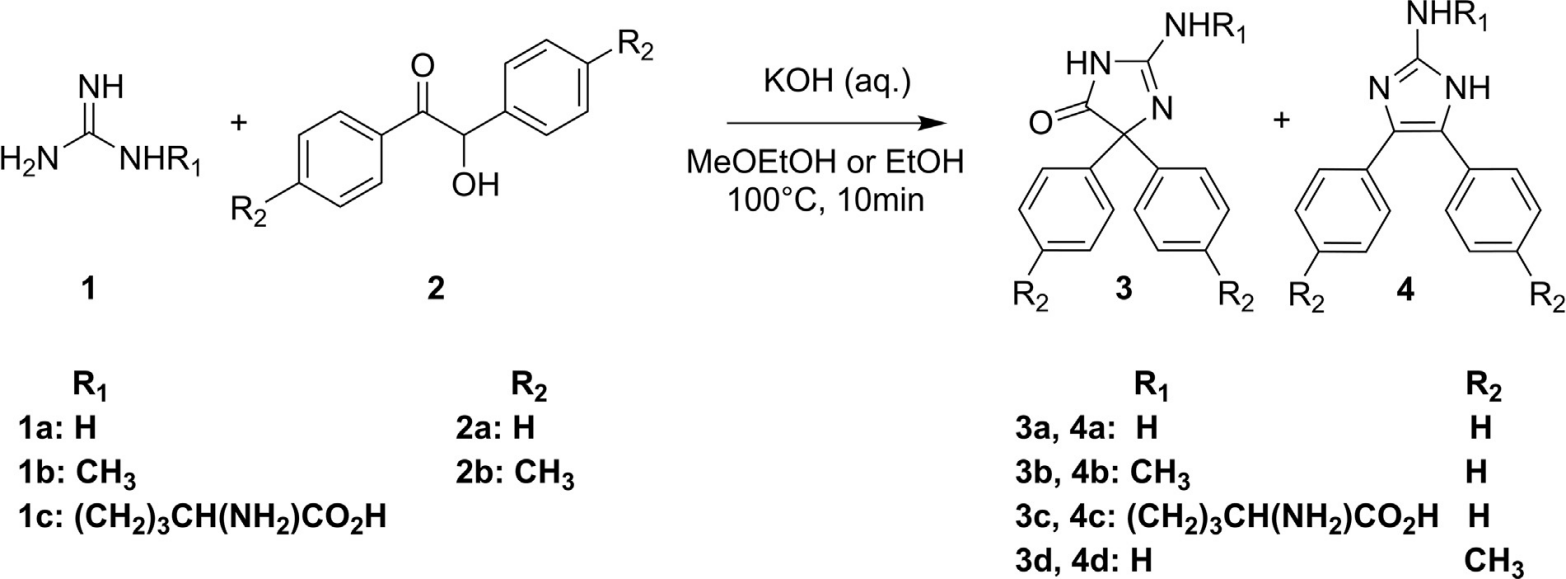
Reaction scheme with all tested variations for the derivatization of guanidino compounds.

**Fig. 2:**
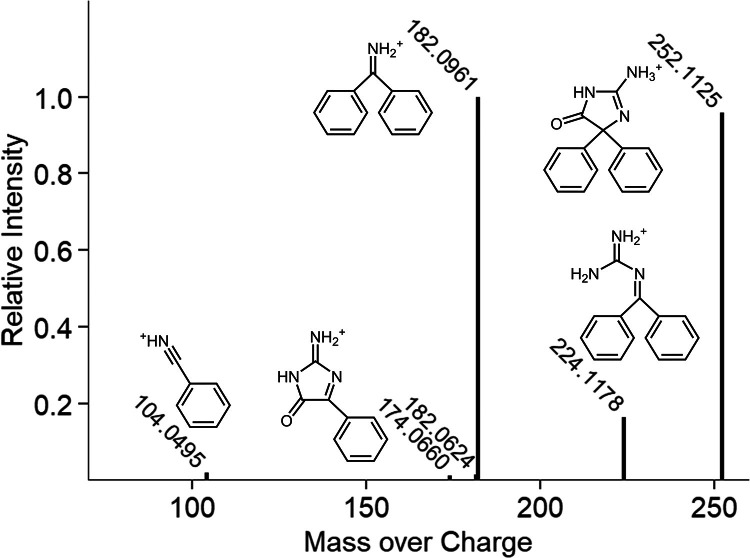
High-resolution product ion spectrum of product **3a** (*m/z*: 252.1125, C_15_H_14_N_3_O^+^, mass deviation −2.53 ppm) with the fragments (*m/z*) 224.1178 (C_14_H_14_N_3_^+^, −1.89 ppm), 182.0961 (C_13_H_12_N^+^, −1.79 ppm), 174.0660 (C_9_H_8_N_3_O^+^, −1.08 ppm), and 104.0495 (C_7_H_6_N^+^, 0.23 ppm). Proposed structures are shown next to the corresponding peak. No structure was proposed for *m/z* 182.0624. Peaks larger than 1 % are shown.

Several studies on guanidine derivatization report the quantification of product **4** (Fig. 1) using fluorescence detection [[6], [7], [8], [9], [10]]. We performed the derivatization as stated by Kai et al. [6]. (i.e., with methoxyethanol as a co-solvent and the use of 75 µL β-mercaptoethanol (0.1 M) and sodium sulfite (0.2 M)). Using mass spectrometry, we detected product **3** at >100 times greater signal intensities compared to product **4**. Product **3** was previously reported as a stable product for a similar derivatization reaction by Fan et al. [11]. They used benzil, an α-diketone, instead of benzoin to derivatize (60°, 4 h) arginine and methylguanidine and quantified product **3** using a liquid chromatography coupled to a quadrupole time-of-flight mass spectrometer. They additionally provide a product ion spectrum for product **3c**. To investigate if we observed the same product as Fan et al., we collected high-resolution mass spectrometry data with a Q Exactive instrument, which was in support of the structure **3a** (Fig. 2). The three most intense fragments were also detected by Fan et al. as the most intense fragments after loss of the side chain R_1_.

We investigated whether variations of the test protocol resulted in different ratios between product **3** and **4** (Fig. 1). First, to test the influence of side chains on product formation, we used different guanidino substrates: guanidine (R_1_ = *H*), methylguanidine (R_1_ = CH_3_) and arginine (R_1_ = (CH_2_)_3_CH(NH_2_)CO_2_H). Second, benzoin (R_2_ = *H*) was replaced with anisoin (R_2_ = CH_3_) to test the influence of the derivatization agent. Third, methoxyethanol was replaced with ethanol to test the influence of the solvent (e.g., different boiling points). All varied protocols resulted in predominantly product **3** (signal intensity of product **4 was** < 1 % of signal intensity of product **3**). Therefore, product **3** can be used to accurately quantify guanidino compounds under all investigated modifications. The varying signal intensities for different substrates and co-solvents (Table 1) were ascribed to different ionization efficiencies. The neglectable difference between ethanol and methoxyethanol as co-solvent enabled us to avoid methoxyethanol, which is a toxic substance, in the final protocol.

**Table 1:** Signal intensities (mean ± SD of triplicates) of product **3** after derivatization of substrates with different derivatization agents and co-solvents.

**Substrate (5µM)**	**Derivatization agent**	**Co-Solvent**	**Retention Time**	**Signal intensity (10^5^) of respective product 3**
Guanidine	Benzoin	Methoxyethanol	3.75	70.7 ± 4.5
Methylguanidine	Benzoin	Methoxyethanol	3.80	110 ± 13
Arginine	Benzoin	Methoxyethanol	3.50	7.0 ± 1.1
Guanidine	Anisoin	Methoxyethanol	3.82	28.8 ± 4.0
Guanidine	Benzoin	Ethanol	3.78	63.9 ± 1.4
Methylguanidine	Benzoin	Ethanol	3.83	72 ± 11
Arginine	Benzoin	Ethanol	3.53	5.58 ± 0.39

Guanidine in environmental matrices is expected to be present in a mixture with other guanidino compounds - for example with the amino acid arginine or the uremic toxin methylguanidine [12]. Such monosubstituted guanidino compounds can interfere in the quantification of guanidine if their side chain is cleaved off during the derivatization. Our protocol was selective for guanidine with interference from methylguanidine and arginine of only 1.1 % and 0.6 %, respectively. These results are in good agreement with the original protocol [6].

### Stability of derivatization product 3

**Fig. 3:**
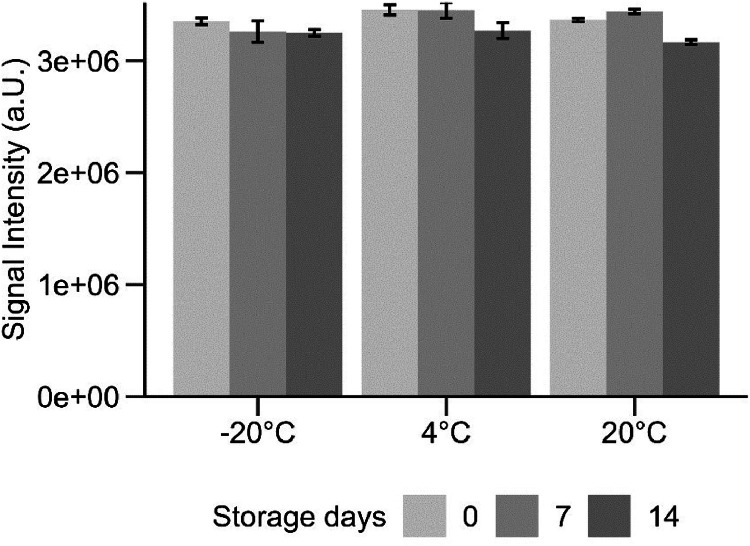
Signal intensity of quantifier ion over time (0, 7, and 14 days) of derivatized guanidine samples (5 µM) at different storage temperatures. Error bars are standard deviation of triplicates.

In the original method, β-mercaptoethanol is added to increase the stability of product **4** [10]. Since product **3** was our predominant product, we tested if β-mercaptoethanol is still necessary to yield a stable product. We quantified 5 µM guanidine samples without the addition of β-mercaptoethanol (as described in the derivatization protocol above) and stored the samples at −20 °C, 4 °C and 20 °C in amber glass vials over 2 weeks. The signal intensity decreased in the storage conditions only by 3 %, 5 %, and 6 %, respectively (Fig. 3). Therefore, we omitted β-mercaptoethanol in the final protocol – resulting in fewer sample preparation steps.

### Limits of detection and quantification

**Fig. 4:**
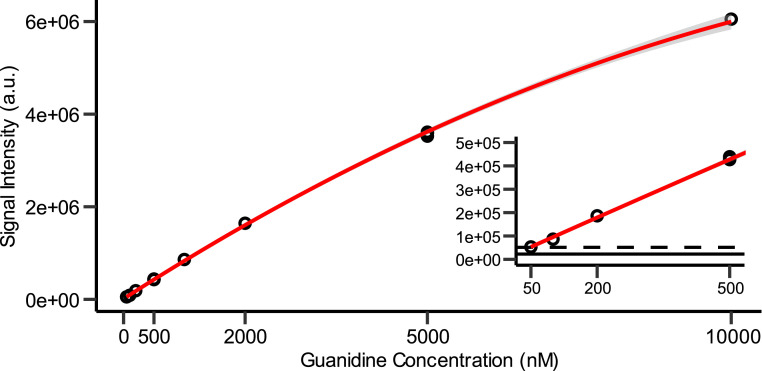
Calibration curve of product **3a:** signal intensity of quantifier ion vs. guanidine concentration for calibration samples. Red line represents a quadratic fit (equation with 1/x weight: *y* = −0.0247x^2^ + 845.3x + 10,998.0, R^2^ = 0.9996). 95 % confidence interval is shown in grey. Limit of detection (LOD) and lower limit of quantification (LLOQ) are shown with solid and dashed black lines, respectively.

We derivatized calibration samples with guanidine concentrations between 50 nM and 10 µM to determine the dynamic range of our method. The best calibration fit was a weighted (1/x) quadratic curve between 50 nM and 10 µM with a correlation coefficient of R^2^ > 0.999 (Fig. 4). The relative standard deviation for triplicates was 1.68 % and 1.54 % for 500 nM and 5 µM, respectively. The limit of detection (LOD) and lower limit of quantification (LLOQ) were calculated with the following formula: $\text{LOD}=\bar{x}+3*\sigma$ and $\text{LLOQ}=\bar{x}+10*\sigma$, where the mean $\left(\bar{x}\right)$ and the standard deviation (σ) were calculated with three individual derivatization blanks (i.e., pure water that was derivatized and quantified). A LOD of 13.7 nM and a LLOQ of 47.9 nM were determined. The lowest calibration standard was chosen in the range of the LLOQ (50 nM). The lowest LLOQ for guanidine with this derivatization technique (Fig. 1) are reported by Gatti et al. using anisoin or fuorin as derivatization agents [7,8]. With anisoin, they report a LLOQ of 0.4 nM (with a calibration sample range from 1.28 to 25.60 µM), while with fuorin, they report a LLOQ of 1 nM (with a calibration sample range from 0.1 to 50.56 µM). Although our LLOQ is considerably higher, to our knowledge we are the first to quantify guanidine concentrations below 100 nM.

**Fig. 5:**
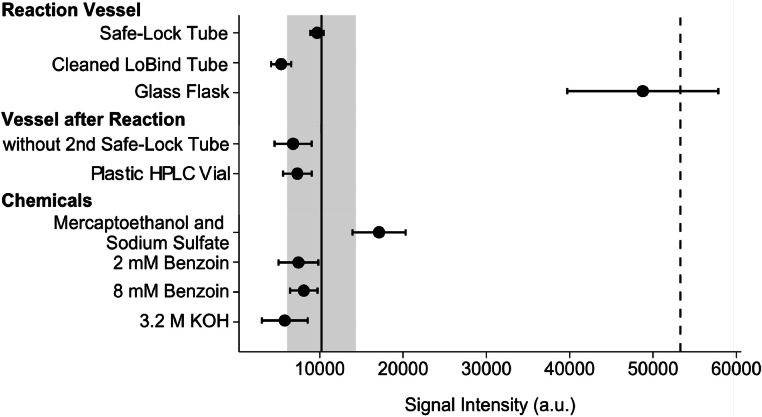
Signal intensity of quantifier ion for derivatization blanks (i.e., no guanidine was added) at varied conditions. Error bars represent standard deviation of triplicates (exception: duplicates for “Glass Flask”). As a reference, the solid vertical line and the grey ribbon represent the mean and standard deviation, respectively, of the final protocol (*n* = 3). The dashed line represents the limit of quantification (50 nM guanidine sample). The reference conditions are as follows: Protein LoBind Tubes, with tube transfer and centrifugation, glass HPLC vials, without β-mercaptoethanol and sodium sulfate, 4 mM benzoin, and 1.6 M KOH. For each test only the indicated condition was changed.

The LLOQ was governed by a constant background signal in the derivatization blank. We varied the derivatization protocol at each step to identify the source of this background signal (Fig. 5). The calibration samples were used as a reference. To test if the background signal originates from the reaction vessel, e.g., by leaching of potentially present guanidine from the vessel during heating, we tested different vessels: Protein LoBind tubes (reference); Safe-Lock tubes; cleaned Protein LoBind tubes (cleaned by one blank derivatization); and to avoid plastic a 50 mL glass round-bottom flask under reflux (which was used with a scaled reaction volume of 30 mL). Although insignificantly (*p* = 0.063), the cleaned Protein LoBind tubes had over 50 % reduction in the background signal. Using the round-bottom flask resulted in a significant (*p* = 0.037) increase in the background signal. We note that the round bottom flask was cleaned before derivatization and the two measurements with this vessel were done consecutively in the same flask. We do not have an explanation for this observation.

Furthermore, we also investigated the role of the vessel type used after the heating step. Therefore, we omitted the centrifugation step which involves a transfer of the derivatization solution to a fresh 1.5 mL Safe-Lock tube. In a separate test, we used plastic HPLC vials instead of glass HPLC vials. Both tests did not reduce the background intensity (*p* > 0.05). In a next step, we considered all chemicals as a contamination source. We changed the concentration of benzoin from 4 mM to 2 mM and 8 mM, and the concentration of KOH from 1.6 M to 3.2 M (the acid concentration was adapted accordingly). These changes did not affect the background intensity (*p* > 0.05). Lastly, we tested if the use of β-mercaptoethanol (used to stabilize product **4**) and sodium sulfite (used to reduce background fluorescence) would reduce the background intensity. With the addition (according to the original protocol [6]) of both chemicals, we detected an increase (+67 %, *p* = 0.044) of the background intensity. Since both chemicals are not necessary for the reliable detection of product **3**, they were not used in the final protocol.

**Fig. 6:**
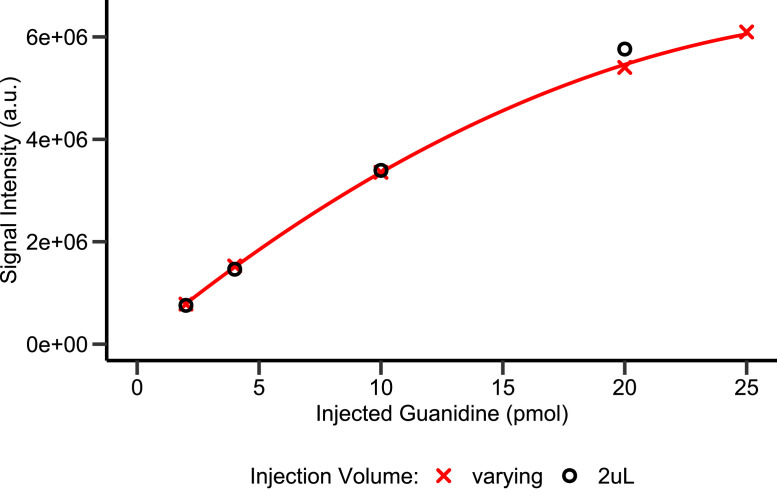
Signal Intensity of quantifier ion vs. amount of injected guanidine for calibration samples with 2 µL injection volume, and a 5 µM calibration sample with varying injection volumes. Red line represents a quadratic fit for the varying injection volume (R^2^ > 0.999).

**Fig. 7:**
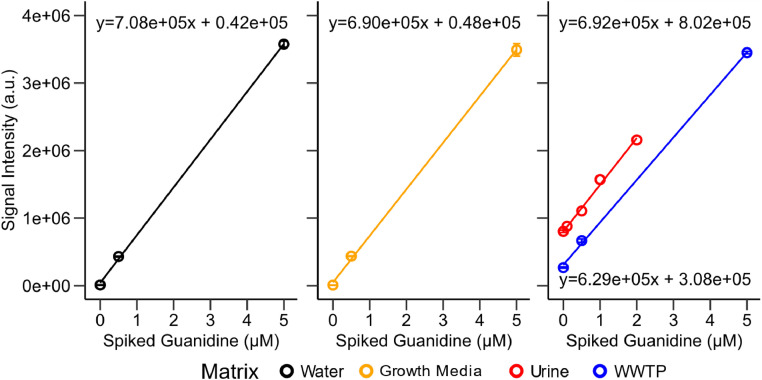
Signal intensity of quantifier ion vs spiked guanidine concentration in different matrices. Error bars represent standard deviation of triplicates. Colored lines represent a linear fit for each matrix, the equation is given in the respective plot. Urine samples were diluted tenfold before quantification.

At the upper end of the calibration range (i.e., at guanidine concentrations above 5 µM; Fig. 4), we observed a quadratic behavior of signal intensity. We assessed this quadratic behavior by varying the injection volume of a 5 µM guanidine calibration sample (Fig. 6). The similarity of the quadratic behavior between this injection-volume-varied curve and the dilution-series calibration curve indicates that the non-linearity is not due to the derivatization step. We hypothesize that this non-linearity is due to saturation of the mass spectrometer at high concentrations [13]. To quantify high guanidine concentrations, samples should thus be diluted with water after derivatization. This was successfully done for urine samples up to a guanidine concentration of 20 µM and in experiments with a biological growth medium up to a concentration of 100 µM (Fig. 7 and related research article).

### Derivatization in environmental matrices

Guanidine is considered as an environmentally relevant nitrogen source and growth substrate [[1], [2], [3], [4], [5]]. It is still largely unknown in which environments guanidine is present and at what concentration. Therefore, we tested if our adapted method can be applied to samples with different matrices. We included human urine and wastewater as samples where guanidine is known or expected to occur [12]. Furthermore, we included a growth medium as a matrix in which microbial guanidine studies are performed. The urine was sampled from a urine collection tank in an office building with urine-separating toilets. When urine is collected, it is diluted a maximum of 2-fold with rainwater and retained in the collection tank for an average of 25 days. The wastewater sample was an influent grab sample from a wastewater treatment plant. The biological growth medium we tested is regularly used for cultivating nitrifiers and contains 4 mM HEPES and high salt concentrations (Growth media composition: 0.95 g/L HEPES, 584 mg/L NaCl, 147 mg/L CaCl_2_, 74.4 mg/L KCl, 54.4 mg/L KH_2_PO_4_, 49.3 mg/L MgSO_4_ × 7 H_2_O, 1 mg/L FeSO_4_ × 7 H_2_O, 80 µg/L CoCl_2_ × 6 H_2_O, 72.6 µg/L Na_2_MoO_4_ × 2 H_2_O, 70 µg/L ZnCl_2_, 50 µg/L H_3_BO_3_, 34.4 µg/L MnSO_4_ × H_2_O, 24 µg/L NiCl_2_ × 6 H_2_O, 20.0 µg/L CuCl_2_ × 2 H_2_O, 4 µg/L Na_2_WO_4_ × 2 H_2_O, 3 µg/L Na_2_SeO_3_ × 5 H_2_O) [14].

The different matrices had only minimal influence on the signal intensity of derivatization product **3a** (Fig. 7). With the use of matrix-matched calibration, our protocol is suitable for all tested matrices. We note that we detected guanidine in urine (11.6–23.2 µM), which is consistent with the literature [8]. In municipal wastewater, we already reported a concentration of 0.49 µM in our related research article [5]. To calculate the theoretical guanidine input from urine in wastewater, we assumed a dilution factor of urine of 65 (2 L urine per person per day [15] and 130 L household effluent per person per day [16]), which results in an estimated concentration of 0.18–0.36 µM. The measured wastewater guanidine concentration is therefore within a reasonable range.

## Limitations

While our adapted method enables detecting guanidine in complex biological and environmental samples down to concentrations in the range of 10 nM, further reduction of this detection limit may be necessary to detect and quantify naturally occurring guanidine in environments such as soil or seawater, which may exhibit low steady-state concentrations. Beyond optimizing derivatization-based methods, an alternative approach could involve the direct analysis of guanidine using mass spectrometry, coupled with front-end separation by hydrophilic interaction liquid chromatography (HILIC) and pre-reduction of high salt concentrations. Lastly, we note that further adaptations of the chromatography method (e.g., using a shorter column) can reduce the required analysis time and the amount of used solvent.

## CRediT author statement

**Richard Gruseck:** Conceptualization, Methodology, Formal analysis, Investigation, Writing – Original Draft, Visualization. **Márton Palatinszky:** Conceptualization, Writing – Review & Editing **Michael Wagner:** Resources, Writing – Review & Editing. **Thilo Hofmann:** Resources, Writing – Review & Editing. **Michael Zumstein:** Conceptualization, Resources, Writing – Original Draft, Review & Editing, Supervision.

## Declaration of competing interest

The authors declare that they have no known competing financial interests or personal relationships that could have appeared to influence the work reported in this paper.

## Data Availability

Data will be made available on request.
